# Bone Scan Index as a prognostic imaging biomarker during androgen deprivation therapy

**DOI:** 10.1186/s13550-014-0058-y

**Published:** 2014-10-17

**Authors:** Mariana Reza, Anders Bjartell, Mattias Ohlsson, Reza Kaboteh, Per Wollmer, Lars Edenbrandt, Elin Trägårdh

**Affiliations:** 1 Division of Clinical Physiology and Nuclear Medicine, Department of Clinical Sciences, Skåne University Hospital, Malmö, Lund University, Inga Marie Nilssons gata 49, Malmö SE-205 02, Sweden; 2 Division of Urological Cancers, Department of Clinical Sciences, Skåne University Hospital, Malmö, Lund University, Lund 22100, Sweden; 3 Department of Astronomy and Theoretical Physics, Lund University, Lund 22100, Sweden; 4 Department of Molecular and Clinical Medicine, Sahlgrenska University Hospital, Gothenburg 413 45, Sweden

**Keywords:** Prostate cancer, Bone Scan Index, Androgen deprivation therapy, Bone metastases

## Abstract

**Background:**

Bone Scan Index (BSI) is a quantitative measurement of tumour burden in the skeleton calculated from bone scan images. When analysed at the time of diagnosis, it has been shown to provide prognostic information on survival in men with metastatic prostate cancer (PCa). In this study, we evaluated the prognostic value of BSI during androgen deprivation therapy (ADT).

**Methods:**

Prostate cancer patients who were at high risk of a poor outcome and who had undergone bone scan at the time of diagnosis and during ADT were recruited from two university hospitals for a retrospective study. BSI at baseline and follow-up were calculated using an automated software package (EXINIbone^bsi^). Associations between BSI, other prognostic biomarkers and overall survival (OS) were evaluated using a Cox proportional hazards regression model.

**Results:**

One hundred forty-six PCa patients were included in the study. A total of 102 patient deaths were registered, with a median survival time after the follow-up bone scan of 2.4 years (interquartile range (IQR) =0.8 to 4.4). Both at baseline and during ADT, BSI was significantly associated with OS in univariate and multivariate analyses. When BSI was added to a prognostic base model including age, prostate-specific antigen, clinical tumour stage and Gleason score, the concordance index increased from 0.73 to 0.77 (*p* =0.0005) at baseline and from 0.77 to 0.82 (*p* <0.0001) during ADT.

**Conclusions:**

Automated BSI during ADT is an independent prognostic indicator of OS in PCa patients with bone metastasis. It represents an emerging imaging biomarker that can be used in a prognostic model for risk stratification of PCa patients at the time of diagnosis and at later stages of the disease. BSI could then help physicians identify patients who could benefit from more aggressive therapies.

##  Background

Early diagnosis of prostate cancer (PCa) has increased in recent decades, and the mortality rate has decreased in countries where the prostate-specific antigen (PSA) blood test was introduced at an early stage [1]. However, in the management of patients at high risk of a poor outcome, there is still an urgent need for improved treatment and monitoring of the disease [1]. While newly diagnosed low-risk patients have shown increasing survival in various studies, patients at high risk of relapse after treatment still show low survival rates, a result strongly associated with metastatic disease [2]. Evaluation of bone metastases is important in risk stratification for optimum decision-making, as many patients should be offered multimodality treatment. In PCa patients with bone metastases, the current standard primary treatment is androgen deprivation therapy (ADT) and careful follow-up involving measurement of serum testosterone and PSA [3]. In time, many patients progress to a castration-resistant phase of the disease (CRPC) with a subsequent need for second-line therapies such as chemotherapy, new androgen-signalling blocking agents and bone-targeting radioisotopes [4].

As several new agents have been presented as treatment options for these patients - with encouraging results - there is an urgent need for predictive tools before initiation of such treatments. It is perhaps even more important to find objective and reproducible tools for evaluation of response and to identify when the treatment no longer has a desired effect. Despite the increasing availability of advanced imaging modalities such as magnetic resonance imaging (MRI) and positron emission tomography/computed tomography (PET/CT), bone scan (BS) is still the most commonly used method of assessing metastatic spread to the skeleton and monitoring response to treatment [5],[6]. It has been used for many years, but there is still no standardised way of describing the images other than in vague terms, i.e. the presence or absence of tumour spread to the skeleton (M1 or M0).

Quantitative measurement of images can provide useful information of clinical relevance, one example being Bone Scan Index (BSI) - an imaging biomarker that more objectively quantifies bone metastases as a percentage of the total skeletal mass affected by metastatic disease [7]. BSI has been shown to contain prognostic information in PCa patients with bone metastases [8], but it has not yet been introduced in a clinical setting, most probably because its calculation is time-consuming and it requires an experienced reader. The recent development of an automated method for calculation of BSI has provided us with a rapid and highly reproducible method of obtaining information on tumour burden, making it more attractive and feasible for routine clinical use [9]. It has been demonstrated that automated BSI correlates well with the manual method and is of prognostic value in men with PCa metastases at the time of diagnosis [9],[10]. The value of a biomarker can vary from one phase of disease to another, as with PSA, which is of greater use in a screening situation and during early phases of disease than in patients with CRPC [11]. Although automated BSI has been proposed as a prognostic biomarker at the time of diagnosis [9],[10], no data is available on its possible prognostic value during ADT. In this study, we evaluated the value of BSI as a biomarker for treatment response during ADT in PCa patients.

##  Methods

###  Patient cohort

We retrospectively studied a series of consecutive PCa patients who had undergone BS as part of the clinical routine at Skåne University Hospital in Malmö from 1996 to 2010 and at Sahlgrenska University Hospital in Gothenburg from 2002 to 2008 and were considered for inclusion. Baseline bone scans of patients from Sahlgrenska University Hospital have also been used in a previous publication [10]. Patients meeting all the following inclusion criteria were selected for the study:

1. At high risk at the time of diagnosis, i.e. at least one of the following criteria in accordance with the European Association of Urology guidelines 2013 was met [6]: clinical tumour stage (cT) T3/T4, biopsy Gleason score (GS) 8 to 10 or PSA concentration in blood >20 ng/mL.

2. Availability of baseline scan: defined as whole-body BS within 3 months of diagnosis and before initiation of ADT.

3. Availability of follow-up scan: defined as whole-body BS during hormonal treatment (>3.0 months after start of treatment).

4. No treatment for PCa prior to baseline BS.

The study was performed in accordance with the Declaration of Helsinki and was approved by the Regional Ethical Review Boards at Lund University and Gothenburg University, Sweden.

###  Bone scan

At both hospitals, whole-body BS was performed 2 to 4 h after intravenous injection of 600 MBq technetium-99m methylene diphosphonate (Tc-99m MDP) (Amersham International plc, Amersham, UK). Anterior- and posterior-view whole-body images were obtained using a gamma camera equipped with low-energy, high-resolution, parallel-hole collimators (MultiSPECT2 (Southern Scientific Ltd., West Sussex, UK) or Siemens Symbia T (Siemens Healthcare Diagnostics Inc. Deerfield, IL, USA) or Maxxus (General Electric Medical Systems, Milwaukee, WI, USA)), using a scan speed of 15 cm/min and a matrix of 256 × 1024. Energy discrimination was provided by a 15% window centred on the 140 keV of Tc-99m.

###  Bone Scan Index calculation

BSI, the measure of the total skeletal mass affected by metastatic disease, was calculated using the software EXINIbone^bsi^ version 1.8 (EXINI Diagnostics AB, Lund, Sweden). The automated method to calculate BSI, which has been described in detail elsewhere [9], consists of four steps. First, the different anatomical regions of the skeleton such as the skull, ribs, vertebra and pelvis are segmented. Second, hotspots are detected and features describing them such as intensity, size, shape and position are calculated. Third, artificial neural networks are used to classify each hotspot as metastatic lesion or not based on the hotspot features. The neural networks have been trained to mimic experienced readers in distinguishing between metastatic lesions and benign hotspots due to, for example, degenerative disease or fracture. Fourth, the BSI is calculated as the sum of volumetric fraction of the skeleton for all hotspots classified as metastatic lesions.

###  Data collection

Retrospectively, clinical data at diagnosis, including age, cT and GS, was collected from the medical records, as well as information on PSA concentration in the blood both at diagnosis and at follow-up. Data on survival was collected from the National Swedish Population Registry. We defined overall survival (OS) as the time from BS to death from any cause. All data collected was anonymised and updated up to 31 December 2013.

###  Statistical analysis

The association between clinical stratification data (age, cT, GS and PSA), BSI and OS was evaluated using the Cox proportional hazards regression models, using both univariate and multivariable modelling. Hazard ratios (HR) together with 95% confidence intervals were estimated, and discrimination between the different survival models was assessed using the concordance index (C-index). The significance of a difference in C-index between different models was calculated using the method described by Haibe-Kains et al. [12].

Kaplan-Meier estimates of the survival function and the log-rank test were used to indicate a significant difference between groups stratified in accordance with the BSI values. In the survival analysis, all data was censored at a follow-up after 5 years. All analyses were carried out using the R statistical computing environment.

##  Results

A total of 146 PCa patients were included, and their clinical-pathological characteristics are presented in Table 1. One hundred two patients out of the 146 died during the follow-up, with a median survival time from the baseline scan of 6 years (interquartile range (IQR) 3.5 to 8.4). The group of 44 men who were still alive had a median follow-up time from baseline BS of 7 years (IQR 6.4 to 11) and from the follow-up BS of 4.7 years (IQR 3.5 to 7.1). The median time between baseline and follow-up BS in the entire group was 2.9 years (IQR 1.5 to 4.4), and patients received ADT at the time of the follow-up scan for a median of 2.2 years (IQR 1.2 to 4.0).

**Table 1:** **Patient characteristics (*N*** **=146)**

**Patient characteristics at baseline**	**Values at baseline**	**Patient characteristics at follow-up**	**Values at follow-up**
Age (years), median (IQR) (*N* =146)	68 (62 to 74)	Age (years), median (IQR) (*N* =146)	71 (65 to 77)
Baseline PSA (ng/mL), median (IQR) (*N* =146)	72 (20 to 187)	Follow-up PSA (ng/mL), median (IQR) (*N* =146)	23 (2.6 to 89)
Baseline BSI (*N* =146)		Follow-up BSI (*N* =146)	
BSI =0 (M0), *N* (%)	84 (57%)	BSI =0 (M0), *N* (%)	55 (37%)
BSI >0 (M1), *N* (%)	62 (43%)	BSI >0 (M1), *N* (%)	91 (63%)
BSI >0, median (IQR)	1.5 (0.2 to 4.2)	BSI >0, median (IQR)	1.7 (0.3 to 4.9)
Clinical T stage (*N* =131)		BSI change from baseline to follow-up (*N* =146)	
T1, *N* (%)	9 (7%)		
T2, *N* (%)	21 (16%)		
T3, *N* (%)	85 (65%)	BSI increased:	
T4, *N* (%)	16 (12%)	High BSI change, *N* (%)	67 (46%)
Gleason score (*N* =135)		BSI unchanged/decreased:	
5, *N* (%)	1 (0.7%)	Low BSI change, *N* (%)	79 (54%)
6, *N* (%)	6 (4.3%)		
7, *N* (%)	48 (36%)		
8, *N* (%)	36 (26%)		
9, *N* (%)	39 (29%)		
10, *N* (%)	5 (4%)		

*N*, number of patients; IQR, interquartile range; BSI, Bone Scan Index; PSA, prostate-specific antigen; M0, absence of metastasis; M1, presence of bone metastasis.

The androgen deprivation therapy administrated to these patients in most cases comprised bicalutamide (Casodex™, AstraZeneca plc, London, UK) or flutamide (Eulexin™, Schering-Plough Corporation, Kenilworth, NJ, USA), with or without a gonadotropin-releasing hormone analogue (GNRH) agonist such as leuprorelin (Eligard™, Enanton™ or Procren™) and goserelin acetate (Zoladex®). Among the 146 patients, 18 (12%) received anti-androgen monotherapy, whereas 128 (88%) received combined androgen blockade.

**Figure 1:**
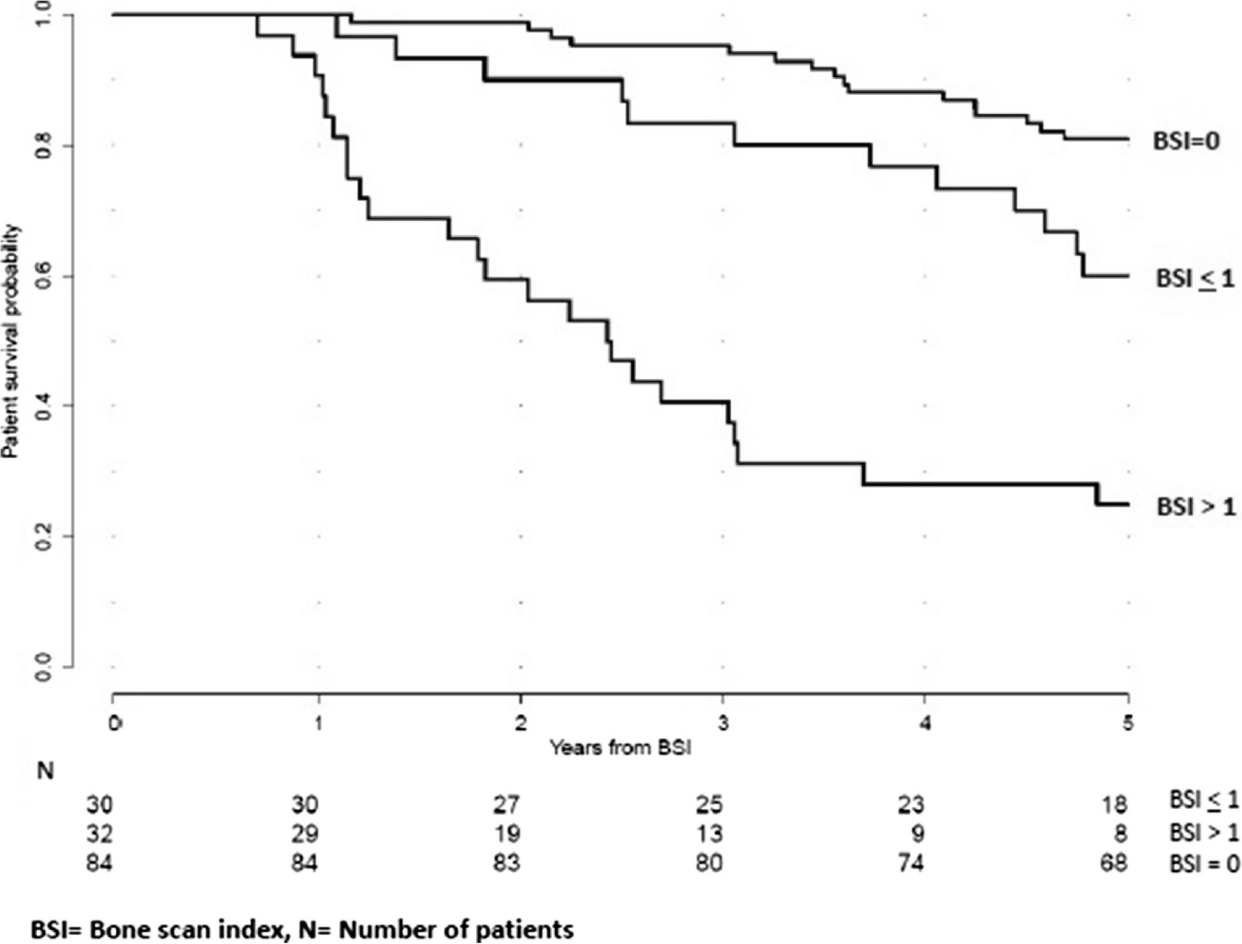
**At baseline, Kaplan-Meier curves showing patient survival probability stratified by BSI categories.** All the 146 patients included in the study were studied at the time of prostate cancer diagnosis with bone scans. In accordance with their BSI values at baseline, these patients were stratified in three BSI categories: BSI =0 (*n* =84), BSI ≤1 (*n* =30) and BSI >1 (*n* =32). These three groups demonstrated significantly different 5-year survival rates of 80%, 60% and 25%, respectively (*p* <0.0001).

Based on previously published data on the prognostic value of BSI, we stratified the patients into three groups based on their baseline BSI: BSI =0 (*n* =84), BSI ≤1% (*n* =30) and BSI >1% (*n* =32), and we could then demonstrate significantly different 5-year survival rates of 80%, 60% and 25%, respectively (*p* <0.0001) (Figure 1).

In the univariate analysis at baseline, BSI, PSA, cT and GS were all significantly associated with OS (Table 2). BSI showed the highest C-index (0.74), followed by GS (0.67). In the multivariate analysis, BSI, cT and GS were associated with OS, while PSA was not. The C-index increased from 0.73 to 0.77 (*p* =0.0005) when adding BSI to a base model including age, PSA, cT and GS.

**Table 2:** Survival analysis demonstrating association between age, PSA, cT, GS and BSI at baseline

**Variable at baseline**	***N***	**Hazard ratio**	***p*** **value**
Univariate analysis			
Age	146	1.02 (0.99 to 1.05)	0.21
PSA	146	1.0002 (1.0000 to 1.0003)	0.02
cT	131	2.40 (1.43 to 4.04)	0.0009
GS	135	1.84 (1.38 to 2.46)	<0.0001
BSI	146	1.27 (1.19 to 1.36)	<0.0001
Multivariate analysis			
Age	121	1.02 (0.98 to 1.06)	0.28
PSA	121	0.9999 (0.9998 to 1.0002)	0.80
cT	121	2.20 (1.28 to 3.78)	0.004
GS	121	1.86 (1.34 to 2.58)	0.0002
Multivariate analysis			
Age	121	1.03 (0.99 to 1.06)	0.10
PSA	121	0.9999 (0.9997 to 1.0002)	0.58
cT	121	1.95 (1.16 to 3.28)	0.01
GS	121	1.99 (1.43 to 2.779)	<0.0001
BSI	121	1.26 (1.16 to 1.37)	<0.0001

PSA, prostate-specific antigen; cT, clinical stage; GS, Gleason score; BSI, Bone Scan Index; *N*, number of patients.

**Figure 2:**
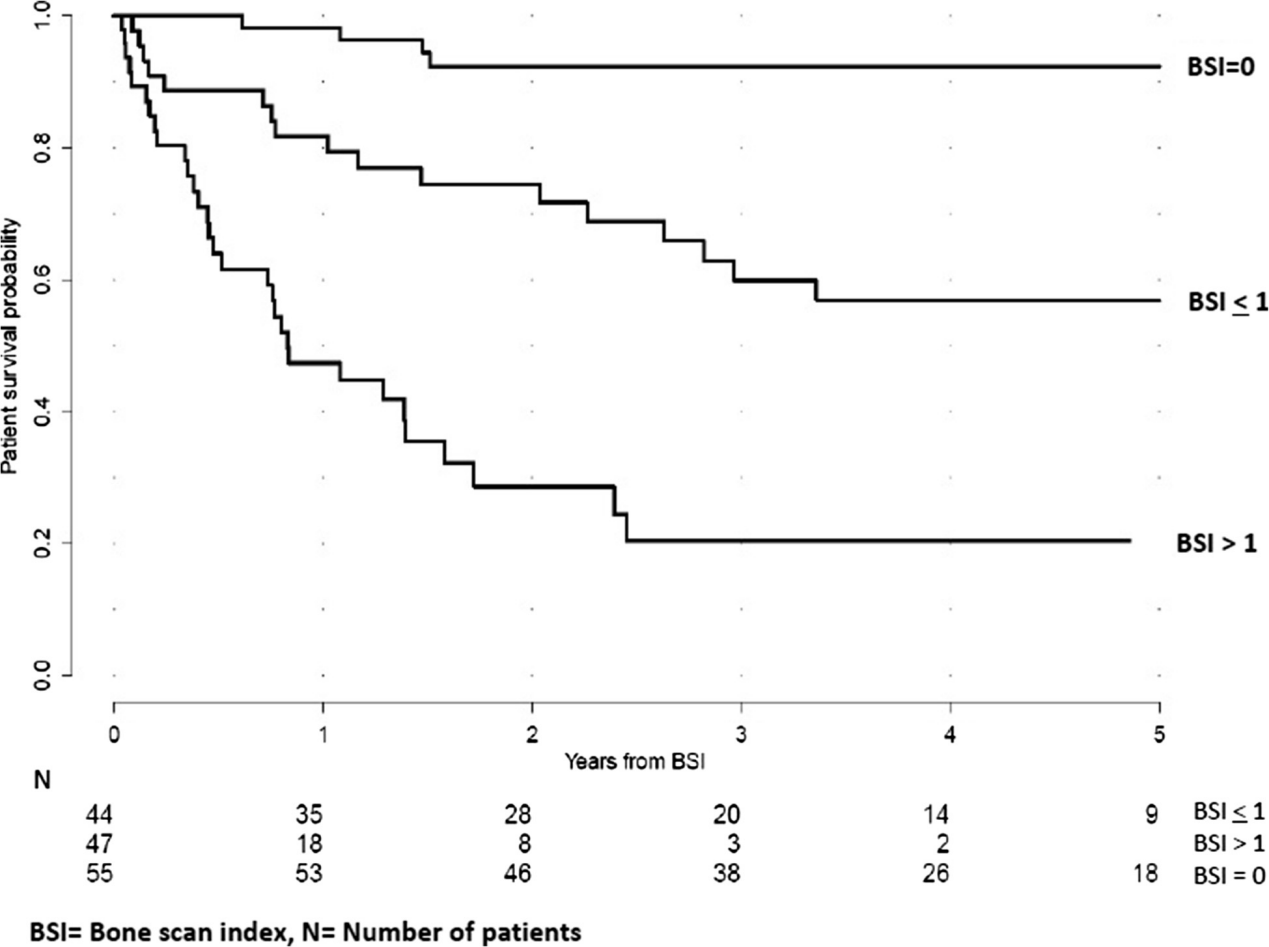
**At follow up, Kaplan-Meier curves showing patient survival probability stratified by BSI categories.** These 146 patients were again studied with bone scans after the initiation of primary hormonal treatment. In accordance with their BSI values at follow-up, these patients were stratified in three BSI categories: BSI =0 (*n* =55), BSI ≤1 (*n* =44) and BSI >1 (*n* =47). These three groups demonstrated significantly different 5-year survival rates of 92%, 57% and 20%, respectively (*p* <0.0001).

Each one of the 146 patients was again retrospectively analysed using a single follow-up BS for measurement of BSI values during ADT. We decided to include only follow-up BS obtained at more than 12 weeks from the start of ADT, as suggested in The Prostate Cancer Working Group 2 (PCWG2) guidelines when evaluating BS after PCa treatment initiation [13]. During ADT, the patients again showed different survival rates depending on their BSI values when we stratified the patients into the same three groups based on their follow-up BSI: BSI =0 (*n* =55), BSI ≤1 (*n* =44) and BSI >1 (*n* =47). These three groups displayed significantly different 5-year survival rates of 92%, 57% and 20%, respectively (*p* <0.001) (Figure 2).

In both univariate and the multivariate analyses at follow-up, we included age, cT and GS at the time of diagnosis and the corresponding follow-up values for BSI and PSA. All these parameters were again associated with OS (Table 3). The C-index for BSI was again the highest (0.80), this time followed by PSA (0.76). The C-index increased from 0.77 to 0.83 (*p* <0.0001) when adding BSI at follow-up to a model including age, cT and GS (at the time of diagnosis) and PSA at follow-up.

**Table 3:** Survival analysis demonstrating association between age, PSA, cT, GS and BSI during ADT

**Variable at follow-up**	***N***	**Hazard ratio**	***p*** **value**
Univariate analysis			
Age	146	1.04 (1.004 to 1.07)	0.03
PSA	146	1.0002 (1.00002 to 1.0003)	0.03
TS	131	2.39 (1.41 to 4.04)	0.001
GS	135	1.75 (1.32 to 2.32)	0.0001
BSI	146	1.26 (1.18 to 1.35)	<0.0001
Multivariate analysis			
Age	121	1.04 (1.0004 to 1.08)	0.03
PSA	121	1.0006 (1.0003 to 1.0009)	<0.0001
TS	121	2.09 (1.24 to 3.51)	0.006
GS	121	1.70 (1.23 to 2.35)	0.001
Multivariate analysis			
Age	121	1.05 (1.01 to 1.09)	0.009
PSA	121	1.0004 (1.00001 to 1.0008)	0.04
TS	121	1.98 (1.21 to 3.24)	0.007
GS	121	1.47 (1.04 to 2.08)	0.03
BSI	121	1.19 (1.09 to 1.29)	<0.0001

PSA, prostate-specific antigen; cT, clinical stage; GS, Gleason score; BSI, Bone Scan Index; ADT, androgen deprivation therapy; *N*, number of patients.

**Figure 3:**
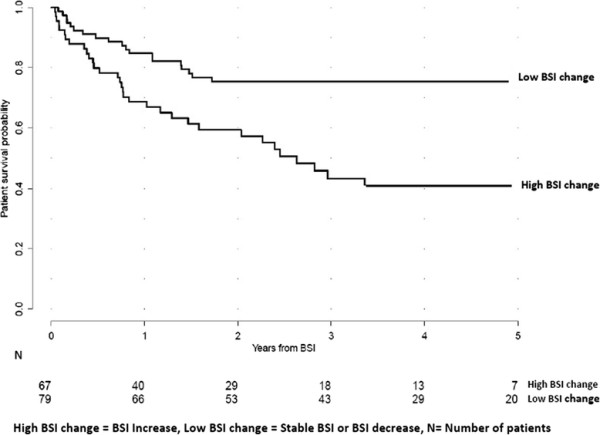
**At follow-up, Kaplan-Meier curves showing patient survival probability stratified by BSI changes categories.** BSI changes from baseline to follow-up were evaluated among the 146 patients studied. In accordance with their BSI change values at follow-up, these patients were classified in two BSI changes categories: High BSI change (BSI increase *n* =67) and low BSI change (stable BSI or BSI decrease, *n* =79). These two groups demonstrated significantly different 5-year survival rates of 41% and 75%, respectively (*p* =0.0004).

Among the 146 patients included in this study, 67 patients showed an increase in BSI from baseline to follow-up (high BSI change). The remaining 79 patients showed a decreased or a stable BSI value (low BSI change). The OS rates were significantly different for these two groups, showing 5-year survival rates of 41% and 75%, respectively (*p* =0.0004) (Figure 3).

## Discussion

While the role of BSI at the time of diagnosis [10],[11] and during chemotherapy [14],[15] has been described in PCa patients, no data is available on the prognostic additive value of BSI in high-risk PCa patients during ADT. Prognostic evaluation of these patients is crucial for optimal multimodality treatment, and our results confirm that the addition of the imaging biomarker BSI as a complement to classical prognostic markers (as PSA, cT and GS) increases the predictive accuracy of risk stratification in this group of patients. Changes in PSA values during treatment constitute one of the most used measures to study outcome in PCa patients, but the isolated measure of this parameter has been shown not to be of prognostic value when corrected by BSI in patients with CRPC who are undergoing chemotherapy [8]. Evaluation of the prognostic additive value of BSI in PCa patients during ADT was thus of interest.

**Figure 4:**
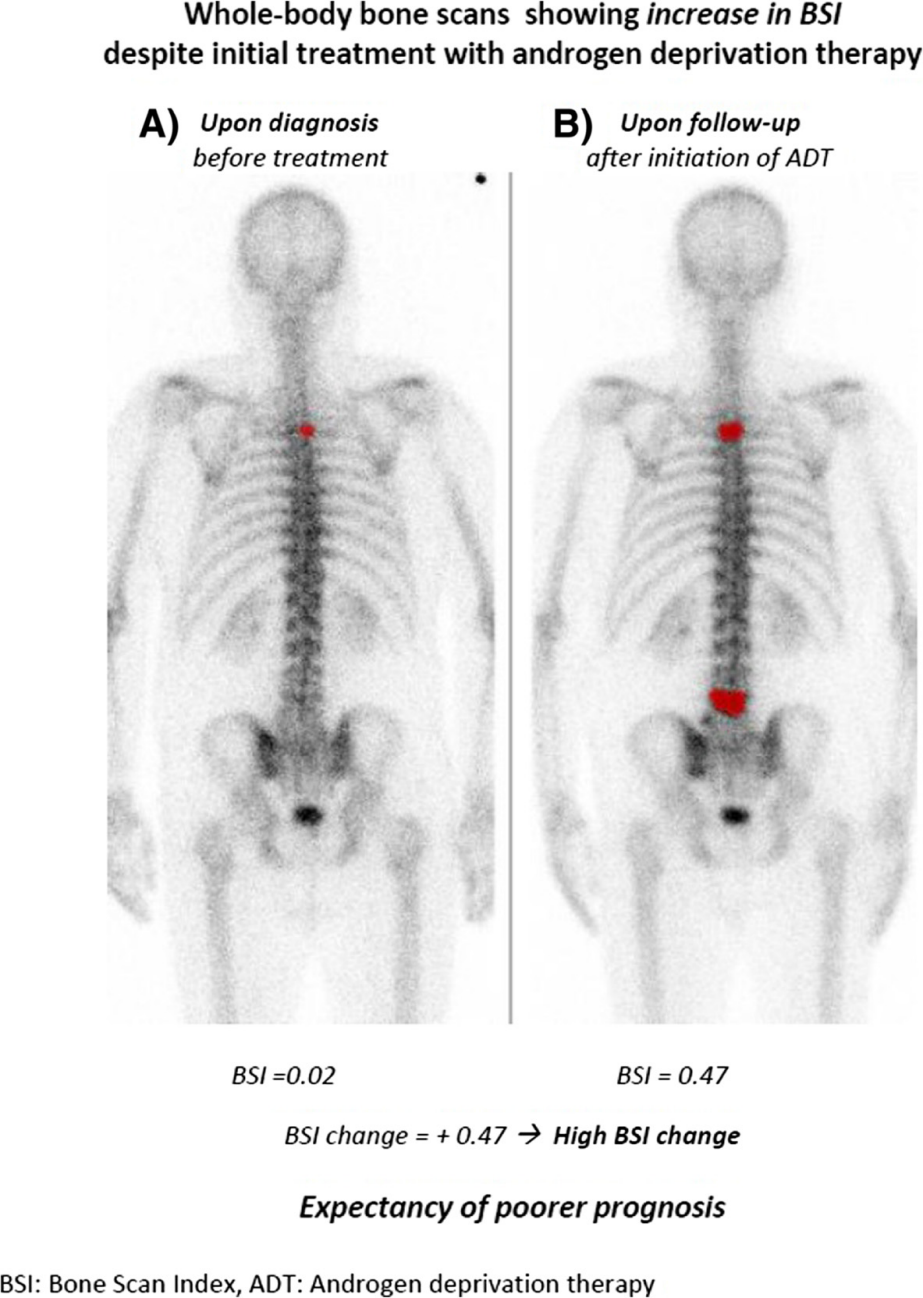
**BSI progression in a metastatic hormone-resistant prostate cancer patient.** This figure shows posterior views of baseline **(A)** and follow-up **(B)** whole-body bone scans of a metastatic hormone-resistant prostate cancer patient. Hot spots, presented in red, represent the bone area affected by tumour. The BSI change from baseline to follow-up shows *BSI progression* in accordance with patient's *high BSI change* after androgen deprivation therapy. According to our results, *high BSI changes* are significantly associated with poorer prognosis.

The results of the present study clearly show that BSI is prognostic for OS during ADT, both as an isolated measure and when added to a classical prognostic model comprising age, PSA, cT and GS. The presence or absence of bone metastases (M1 or M0) is commonly used to risk stratify PCa patients. We also showed that BSI, as a quantitative measurement of the total tumour burden in the skeleton, can be used to further stratify high-risk PCa patients with metastases. Patients with BSI <1 had higher 5-year survival rates compared to those with BSI >1, both at the time of diagnosis and during ADT (Figures 1 and 2). PCa patients with distant metastases at the time of diagnosis most commonly receive ADT as primary treatment. The clinical implication of the findings in our study is that BSI could be used as a complement to conventional prognostic biomarkers in the stratification of high-risk PCa patients in clinical routine and in the design of clinical trials, not only at the time of diagnosis but also during ADT. BSI could then help physicians identify patients who could benefit from more aggressive therapies (Figure 4).

In the present study, we have used OS as an endpoint for outcome analysis. Prostate cancer specific survival would also have been important to evaluate, but due to limited availability of data, this endpoint could not be used. In future prospective BSI studies, we plan to investigate additional endpoints besides OS such as biochemical recurrence, clinical progression and radiographic progression. It would be also of value to evaluate the correlation of BSI with other prognostic biomarkers that showed impact on survival in PCa patients. Therefore, beside the previous studied parameters, we intend to include alkaline phosphatase, haemoglobin, lactate dehydrogenase and performance status in the design of our future prospective studies.

We have retrospectively evaluated a consecutive cohort of prostate cancer patients who underwent whole-body scan examinations both at the time of diagnosis and later on at follow-up after hormonal therapy according to the standard-of-care procedure. At our centres, the indications for bone scan examinations in prostate cancer patients are mainly PSA in blood >20 and/or high GS (>8), increasing PSA values (biochemical progression) and bone pain (symptomatic progression). These indications may increase the possibility of finding bone metastasis already at the time of diagnosis and or progression of disease at follow-up in patients who underwent whole-body scan examinations at our nuclear medicine departments, but to avoid the risk of selection bias, this patient material represents a consecutive cohort.

BSI is advantageous in that it is based on BS examinations, which constitute the most widely used method of evaluating metastatic spread to the bone in PCa patients, as considered to be a golden standard [6]. More advanced imaging modalities such as MRI and PET/CT are still not commonly used and they are, to some extent, hampered by the lack of standardization. A disadvantage of BSI is the limitations of the BS technique itself, e.g. the difficulty of distinguish between flare reactions and disease progress after initiation of treatment. This is most common during the first 3 months of treatment, and we thus decided only to include patients who had undergone baseline scans before initial treatment as well as follow-up scans more than 12 weeks after commencement of ADT, as suggested in the PCWG2 guidelines when evaluating BS after PCa treatment initiation [13]. Despite these cautions to reduce the probability of flare effect during the study design, as well as the fact that there were no signs suggesting its presence in the visual evaluation of the BS images studied (an increase in activity of previously seen metastatic lesions and no new lesions), we cannot ensure the total exclusion of the phenomenon in this material. At present, there is no method that can effectively ensure its exclusion at follow-up. Inclusion of a flare reaction would weaken the association between BSI and OS, since patients with a positive response to treatment and a falsely elevated BSI would most probably have a better prognosis.

BS imaging is a very sensitive technic for osteoblastic bone reactions. An intense uptake in a BS does not always correlate with metastatic disease. These changes can be also found in benign pathologies such as osteomyelitis and fractures. BSI refers to percentage of skeleton affected only by tumour; we have therefore reviewed each BSI measurement and corrected manually those lesions of benign nature that were misclassified as hotspots. This correction though was only needed in less than 5% of the cases.

In the process of implementing BSI as an imaging biomarker for PCa patients, we here add new and important information on the value of BSI during ADT. Further studies on BSI as a clinically useful biomarker to predict and to evaluate response to novel treatments in CRPC are also underway. BSI studies could be of great value to stratify patients in clinical trials and to measure clinical efficacy of new treatments.

## Conclusions

We conclude that BSI during ADT is independently associated with OS. This new imaging biomarker can be used as a complement to conventional prognostic biomarkers such as PSA, GS and cT to stratify high-risk prostate cancer patients, not only at the time of diagnosis but also during later stages of the disease. BSI could then help physicians identify patients who could benefit from more aggressive therapies.

## Competing interests

LE and MO are employed by and are shareholders in EXINI Diagnostics AB (Lund, Sweden), which provides the EXINIbone^bsi^ software used in this study. AB has been receiving honoraria from EXINI Diagnostics as Medical Adviser. MR, RK, ET and PW indicated no potential conflicts of interest.

## Authors’ contributions

MR, AB, PW, LE and ET participated in the design of the study and in the analysis and interpretation of data, and drafted the manuscript. RK participated in the design of the study and in the analysis and interpretation of data. MO carried out the statistical analysis of the data. All authors read and approved the final version of the manuscript.
